# Elemental compositions of lichens from Duolun County, Inner Mongolia, China: Origin, road effect and species difference

**DOI:** 10.1038/s41598-017-06027-z

**Published:** 2017-07-17

**Authors:** Hua-Jie Liu, Jing-Gong Wang, Yu Xia, Meng-Jie Yang, Si-Wa Liu, Liang-Cheng Zhao, Xiu-Ping Guo, Yun-Jun Jiang, Xin Li, Qing-Feng Wu, Shi-Bo Fang

**Affiliations:** 1 College of Life Sciences, Hebei University, Baoding, Hebei 071002 China; 2 Hebei Geological Laboratory, Baoding, Hebei 071051 China; 3 Duolun County Grassland Management Station, Xilinhot, Inner Mongolia 027300 China; 4 State Key Laboratory of Severe Weather, Chinese Academy of Meteorological Sciences, Beijing, 100081 China; 5 Collaborative Innovation Center on Forecast and Evaluation of Meteorological Disasters, Nanjing University of Information Science & Technology, Nanjing, 210044 China

## Abstract

To assess the response of lichen elemental compositions to road traffic and species difference in the context of high dust input and anthropogenic emissions, two foliose epiphytic lichens (*Phaeophyscia hirtuosa*, PHh; *Candelaria fibrosa*, CAf) were sampled near a road adjacent to Dolon Nor Town (Duolun County, Inner Mongolia, China). Twenty elements (Ba, Ca, Cd, Co, Cr, Cu, Fe, K, Mg, Mn, Mo, Na, Ni, P, Pb, Sb, Sr, Ti, V and Zn) in lichen and surface soil samples were analysed using inductively coupled plasma mass spectrometer (ICP-MS). The results demonstrate that lichen elemental compositions are highly influenced by both their natural environment and anthropogenic input. Windblown dust associated with sand dunes and degraded/desertified steppes represents the predominant source of lichen elements. Road traffic can enhance the lichen elemental burden by increasing the number of soil particles. Anthropogenic emissions from the town and road traffic have also led to the enrichment of Cd and Zn in lichens. PHh was higher than CAf in concentrations of 14 terrigenous metals. Both lichens are applicable to biomonitoring of atmospheric element deposition and, in most cases, yield comparable results.

## Introduction

In recent decades, air quality worsened due to frequent dust storms and air pollution in North China. This is particularly true for Duolun County, Inner Mongolia, which has experienced desert expansion, degradation/desertification of steppes, and increasing industrialisation during the last several decades. However, atmospheric element deposition is not fully understood due to the disadvantages of traditional (instrumental) methods, which are low in spatial sampling density and are limited in number of pollutants monitored (mainly CO, SO_X_, NO_X_ and dust)^1, 2^. It is well-known that the lichen biomonitoring technique can be an alternative to traditional methods for this purpose^3, 4^.

Lichens, also known as lichenised fungi, are dependent mainly on atmospheric deposition for mineral nutrients and are good accumulators of trace elements^3–5^. These features, combined with their widespread distribution, slow growth rate, and long life, rank them one of the best biomonitors of air pollution in terrestrial ecosystems^3–5^. The lichen biomonitoring technique is well-established and has been used to monitor atmospheric deposition of trace elements in many countries^1–30^. Studies in two regions of China, the Taihang Mountains of Hebei and the Xilin River Basin of Inner Mongolia, suggest that this technique can distinguish atmospheric inputs from terrigenous inputs and is a useful tool in biomonitoring air pollution^31–34^. These studies also noted that the response of lichen elemental composition to air pollution depends on lichen species^32, 33^. However, these studies were conducted in sites far away from roads or in remote regions. The response of lichen elemental composition to road traffic in the context of high dust input and anthropogenic emissions are yet unknown in Inner Mongolia.

**Figure 1:**
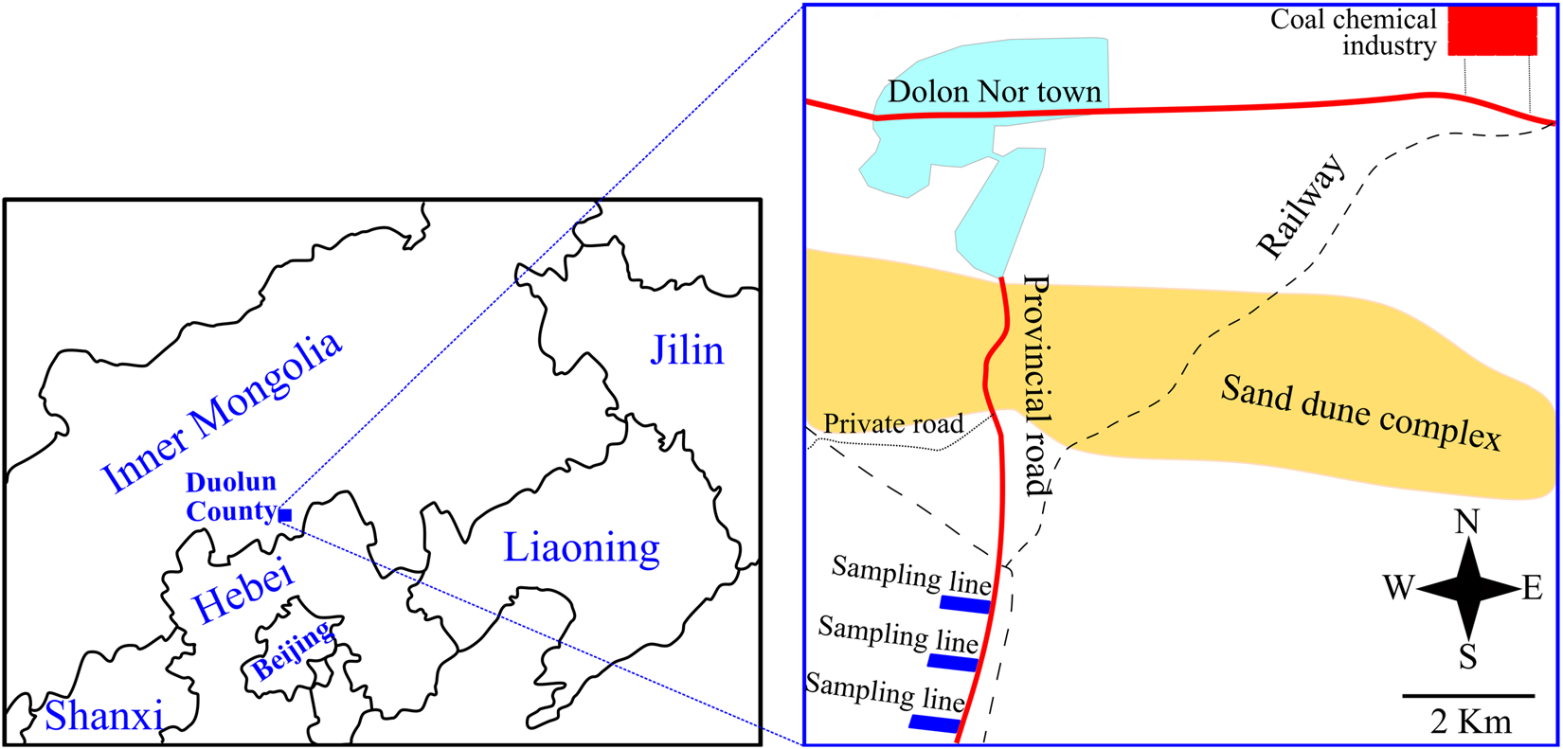
Diagrams showing the location of Duolun County in 2011, and sampling lines. The diagrams were prepared using Inkscape software 0.91 (Free Software Foundation Inc., USA; http://www.inkscape.org/).

We sampled two epiphytic, foliose lichens (*Phaeophyscia hirtuosa*, PHh; *Candelaria fibrosa*, CAf) near a road crossing a forest close to Dolon Nor town, one of the most representative areas of increasing industrialised and degraded/desertified steppe zones in Inner Mongolia (Fig. 1). Twenty elements (Ba, Ca, Cd, Co, Cr, Cu, Fe, K, Mg, Mn, Mo, Na, Ni, P, Pb, Sb, Sr, Ti, V and Zn) were measured. The purposes of the present study were to assess the response of lichen elemental composition to road traffic and species differences by measuring concentrations and identifying element provenance.

## Result

### Elemental concentration, enrichment factor (EF) and Fe:Ti ratio

Mean values with coefficient of variance (CV, defined as the ratio of standard deviation values to mean concentration values) and median values with Interquartile Range (IR, defined as the difference between percentile 75% and percentile 25%) of the element concentration and Fe:Ti ratios are given in Table 1 for 4 data sets (lichen combined, LC; CAf; PHh; and surface soil, SS). The mean concentration values were distributed in the following order in the LC data set: Fe > K > Ca > Na > P > Ti > Mg > Mn > Cr > Zn > Ba > Sr > Cu > V > Pb > Ni > Mo > Co > Sb > Cd. This order was followed in each lichen, except K > Fe in CAf and V > Cu in PHh (Table 1). In each lichen, most elements had a low variation (CV < 25%) in concentration, 4 metals (Cr, Ni, Mg and Mo) had a CV ranging from 25 to 43% (Table 1).

**Table 1:** Elemental concentration, EF_SS_ and Fe:Ti ratio in lichens and surface soil from a *Populus* forest, near a provincial road in Duolun County, Inner Mongolia, China.

Element	Statistics	Concentration (ug·g^−1^)				EF_SS_		
		LC	PHh	CAf	SS	LC	PHh	CAf
Ba	Mean (CV%)	98.93 (21.30)	116.7 (12.76)A	81.19 (5.49)B	585.7 (3.27)	0.14 (8.22)	0.14 (8.30)	0.14 (8.28)
	Median (IR)	92.36 (35.64)	117.7 (20.09)	82.01 (6.51)	590.6 (14.60)	0.14 (0.02)	0.14 (0.01)	0.14 (0.02)
Ca	Mean (CV%)	3524 (23.38)	4209 (10.48)A	2840 (15.85)B	3307 (28.60)	0.88 (9.84)	0.91 (7.42)	0.85 (11.27)
	Median (IR)	3395 (1245)	4281 (682.2)	3036 (703.8)	3039 (736.0)	0.88 (0.11)	0.91 (0.10)	0.86 (0.15)
Cd	Mean (CV%)	0.41 (10.37)	0.42 (9.53)A	0.40 (10.51)A	0.06 (21.90)	6.20 (22.15)	5.37 (13.92)	7.03 (19.47)
	Median (IR)	0.41 (0.05)	0.41 (0.05)	0.40 (0.06)	0.06 (0.02)	5.72 (1.72)	5.45 (0.72)	7.05 (2.27)
Co	Mean (CV%)	2.97 (27.29)	3.58 (18.75)A	2.35 (12.99)B	7.93 (9.92)	0.31 (9.17)	0.32 (9.37)	0.29 (6.67)
	Median (IR)	2.92 (1.04)	3.26 (0.85)	2.22 (0.46)	8.06 (1.16)	0.30 (0.03)	0.31 (0.05)	0.29 (0.03)
Cr	Mean (CV%)	209.9 (28.97)	213.0 (25.79)A	206.7 (33.12)A	392.1 (38.25)	0.45 (28.15)	0.38 (22.52)	0.51 (25.28)
	Median (IR)	178.7 (94.33)	178.7 (86.92)	184.9 (117.3)	404.2 (294.2)	0.41 (0.24)	0.33 (0.09)	0.52 (0.24)
Cu	Mean (CV%)	19.77 (19.84)	22.63 (13.38)A	16.91 (13.40)B	17.67 (11.00)	0.94 (19.68)	0.92 (19.49)	0.96 (20.46)
	Median (IR)	18.59 (6.05)	23.68 (5.62)	17.74 (3.71)	17.70 (4.07)	0.97 (0.29)	0.94 (0.29)	0.98 (0.38)
Fe	Mean (CV%)	9088 (20.31)	10573 (11.79)A	7604 (11.47)B	7497 (24.09)	1.00 (0.00)	1.00 (0.00)	1.00 (0.00)
	Median (IR)	9148 (2267)	10097 (1233)	7830 (1519)	7072 (1509)	1.00 (0.00)	1.00 (0.00)	1.00 (0.00)
K	Mean (CV%)	8526 (7.65)	8655 (7.22)A	8397 (8.10)A	20610 (3.94)	0.35 (19.83)	0.30 (11.21)	0.41 (13.55)
	Median (IR)	8326 (1104)	8694 (783.1)	8103 (867.8)	20420 (630.3)	0.34 (0.12)	0.29 (0.05)	0.42 (0.08)
Mg	Mean (CV%)	463.8 (38.09)	589.1 (24.41)A	338.6 (30.07)B	1361 (43.62)	0.28 (30.80)	0.31 (27.35)	0.25 (31.47)
	Median (IR)	479.2 (246.6)	576.2 (103.3)	329.5 (171.2)	1108 (485.4)	0.28 (0.12)	0.30 (0.08)	0.23 (0.13)
Mn	Mean (CV%)	300.6 (20.28)	331.2 (15.58)A	269.9 (20.46)B	148.3 (32.28)	1.68 (13.25)	1.58 (9.63)	1.79 (13.47)
	Median (IR)	286.4 (97.79)	331.6 (92.19)	255.4 (85.01)	138.9 (62.89)	1.65 (0.25)	1.58 (0.23)	1.69 (0.44)
Mo	Mean (CV%)	4.36 (40.48)	4.68 (38.45)A	4.04 (43.25)A	43.86 (14.76)	0.08 (34.71)	0.07 (34.08)	0.09 (34.34)
	Median (IR)	3.62 (3.25)	3.73 (3.03)	3.37 (2.90)	40.93 (9.88)	0.07 (0.04)	0.06 (0.04)	0.08 (0.05)
Na	Mean (CV%)	1841 (21.49)	2136 (15.58)A	1547 (10.76B)	11298 (3.27)	0.13 (8.17)	0.13 (7.47)	0.14 (9.07)
	Median (IR)	1761 (596.9)	2175 (370.6)	1578 (147.4)	11166 (187.6)	0.13 (0.02)	0.13 (0.02)	0.14 (0.02)
Ni	Mean (CV%)	11.78 (29.00)	13.46 (23.05)A	10.10 (29.08)B	359.7 (8.97)	0.03 (18.67)	0.03 (16.61)	0.03 (20.93)
	Median (IR)	10.97 (5.80)	11.92 (5.58)	8.87 (4.03)	351.7 (20.59)	0.02 (0.01)	0.03 (0.01)	0.02 (0.01)
P	Mean (CV%)	1475 (15.27)	1531 (13.86)A	1419 (16.41)A	108.60 (35.41)	11.59 (23.83)	10.09 (16.67)	13.09 (21.87)
	Median (IR)	1429 (416.4)	1525 (362.3)	1343 (299.8)	86.15 (49.13)	11.26 (3.78)	9.93 (2.63)	13.17 (4.16)
Pb	Mean (CV%)	13.35 (23.51)	15.93 (14.22)A	10.78 (9.19)B	13.43 (15.07)	0.82 (8.31)	0.84 (8.30)	0.79 (7.56)
	Median (IR)	13.09 (4.75)	15.01 (2.85)	10.26 (1.76)	12.37 (3.17)	0.81 (0.09)	0.85 (0.09)	0.79 (0.07)
Sb	Mean (CV%)	0.49 (23.94)	0.58 (18.36)A	0.41 (13.02)B	0.43 (9.69)	0.95 (7.27)	0.95 (8.74)	0.94 (5.81)
	Median (IR)	0.49 (0.13)	0.53 (0.09)	0.40 (0.08)	0.42 (0.04)	0.95 (0.11)	0.94 (0.13)	0.95 (0.09)
Sr	Mean (CV%)	44.55 (24.64)	53.09 (16.15)A	36.01 (12.22)B	146.3 (3.56)	0.25 (8.11)	0.26 (6.90)	0.24 (8.77)
	Median (IR)	43.75 (16.53)	51.31 (12.95)	34.78 (5.52)	145.2 (5.15)	0.25 (0.03)	0.26 (0.03)	0.24 (0.03)
Ti	Mean (CV%)	720.6 (23.42)	861.3 (12.89)A	579.8 (10.88)B	631.7 (36.56)	0.94 (5.48)	0.97 (3.94)	0.91 (5.07)
	Median (IR)	707.2 (243.2)	825.1 (110.7)	581.9 (110.3)	546.9 (223.7)	0.94 (0.08)	0.98 (0.07)	0.91 (0.07)
V	Mean (CV%)	19.10 (21.03)	22.75 (8.01)A	15.45 (7.54)B	16.31 (22.57)	0.97 (6.32)	0.99 (5.82)	0.94 (5.64)
	Median (IR)	18.51 (6.65)	22.44 (1.60)	15.79 (1.91)	15.49 (3.41)	0.95 (0.09)	0.99 (0.09)	0.94 (0.06)
Zn	Mean (CV%)	125.8 (8.89)	127.5 (10.83)A	124.0 (6.44)A	16.96 (27.07)	6.34 (21.08)	5.36 (11.06)	7.32 (15.58)
	Median (IR)	124.8 (13.53)	125.2 (21.23)	123.7 (5.42)	14.59 (6.66)	6.26 (1.59)	5.35 (0.74)	6.94 (1.13)
Fe:Ti	Mean (CV%)	12.71 (5.63)	12.30 (3.97)B	13.12 (5.19)A	12.27 (10.73)			
	Median (IR)	12.59 (1.03)	12.17 (0.89)	13.00 (1.04)	12.69 (1.58)			

Different capitalised letters in each row indicate a significant difference in element concentration between the two lichens (p ≤ 0.05) based on Paired-Samples T test performed on the raw concentrations. CV refers to the coefficient of variance (defined as the ratio of standard deviation values to mean concentration values). IR refers to the interquartile range (defined as the difference between percentile 75% and percentile 25%). EF_SS_ refers to the enrichment factor normalised to the local surface soil with Fe as a reference element according to Equation 1. LC refers to lichen combined (n = 24). PHh refers to *Phaeophyscia hirtuosa* (n = 12). CAf refers to *Candelaria fibrosa* (n = 12). SS refers to surface soil (n = 9).

A one-sample Kolmogorov-Smirnov test performed on the raw concentrations demonstrated normal distributions for all elements in each data set (all P > 0.05), dictating the use of the parametric test in subsequent statistical analyses. Pearson correlation analyses show that Fe and Ti were highly correlated in each of the four data sets (all r > 0.979, p < 0.001). Independent sample T tests show that the Fe:Ti ratios in LC (12.71), PHh (12.30) and CAf (13.12) were closely similar to that in SS (12.27; p > 0.05; Table 1).

**Figure 2:**
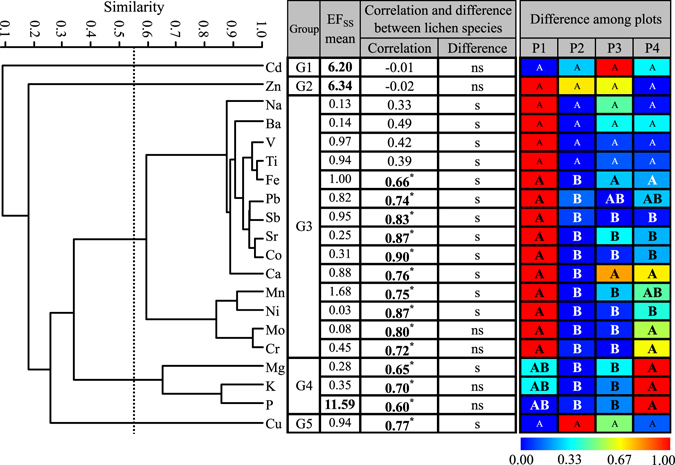
Dendrogram obtained using UPGMA cluster analysis on the correlation distance matrix (left panel) and table showing EF_SS_ and elemental concentration relationships between lichen species and among plots (right panel). The broken line in the dendrogram shows a correlation similarity of 0.55. In the Correlation column, “*” denote correlation between lichens are significant at p < 0.05. In the Difference column, “ns” and “s” denote insignificant (p > 0.05) and significant (p ≤ 0.05) difference between lichens, respectively. In the Difference among plots columns, different capitalised letters in a row indicate a significant difference in element concentration among plots. Different background colours in each row are the heatmap showing range-standardised concentrations. EF_SS_ denotes enrichment factor normalised to local surface soil with Fe as a reference element according to Equation 1. P1, P2, P3 and P4 denote plots with a distance from the road of 5–10 m, 100 m, 200 m and 400 m, respectively. Cluster analysis was performed on the raw concentrations (n = 24), and the resultant cophenetic correlation index is 0.896. Concentration correlation and difference between lichen species were performed on raw concentrations using Pearson correlation test and Paired-samples T test, respectively. Concentration differences between plots were evaluated using Paired-samples T tests, followed by a Bonferroni correction (α = 0.0085). The heatmap was generated using Past 3.14 software (Ø. Hammer, Sep. 2016). All tests were performed on *Candelaria fibrosa* (n = 12) and *Phaeophyscia hirtuosa* (n = 12).

According to Equation 1, element concentrations in the lichens were normalised to SS with Fe as a reference element to obtain EF_SS_. Three elements (Cd, P and Zn) in lichens had an EF_SS_ of >5.0. Other elements had an EF_SS_ of <1.8 (Table 1). These results are also presented in Fig. 2.

### Concentration correlation

An UPGMA (unweighted pair-group method with arithmetic means) cluster analysis was performed on a correlation matrix of the LC, CAf and PHh data sets separately. The results show that concentration correlations between lichen elements are well presented by cluster analyses, which preserved most of the pairwise distances between the original unmodeled data points (cophenetic correlation coefficient: LC, 0.896; CAf, 0.921; PHh, 0.919).

**Figure 3:**
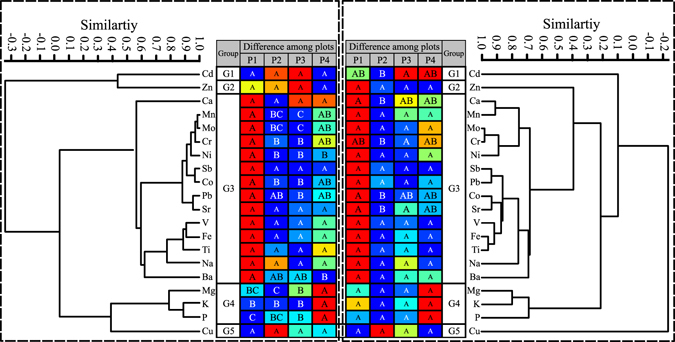
Dendrograms obtained using UPGMA cluster analysis on the correlation distance matrix, and table showing elemental concentration differences among plots for CAf (**a**) and PHh (**b**). The broken line in the dendrogram shows a correlation similarity of 0.55. In the Difference among plots rows, different capitalised letters in a row denote a significant difference in element concentration among plots. Different background colours in each row are the heatmap showing range-standardised concentrations. P1, P2, P3 and P4 denote plots with a distance to the road of 5–10 m, 100 m, 200 m and 400 m, respectively. Cluster analysis was performed on the raw concentrations, and the resultant cophenetic correlation index was 0.921 for CAf and 0.919 for PHh. Concentration differences between plots were evaluated using Paired-samples T tests followed by a Bonferroni correction (α = 0.0085). The heatmap was produced using Past 3.14 software (Ø. Hammer, Sep. 2016). CAf refers to *Candelaria fibrosa* (n = 12). PHh refers to *Phaeophyscia hirtuosa* (n = 12).

Generally, the concentration correlations observed in LC (Fig. 2) were closely similar to those in CAf (Fig. 3a) and PHh (Fig. 3b). In all three data sets, elements can be classified into 5 groups at a correlation similarity of 0.55 (Figs 2 and 3). Cd in group G1, Zn in G2 and Cu in G5 had a poor correlation with other elements. G4 contains K, P and Mg (Figs 2 and 3), which are significantly and positively intercorrelated (K-P: r = 0.856 and p = 0.001 in LC, r = 0.896 and p < 0.001 in CAf, r = 0.793 and p = 0.002 in PHh; Mg-P: r = 0.623 and p = 0.001 in LC, r = 0.778 and p = 0.003 in CAf, r = 0.591 and p = 0.003 in PHh). The other 14 elements belong to G3 (Fig. 2).

PHh and CAf are positively correlated for 14 elements (Ca, Co, Cr, Cu, Fe, K, Mg, Mn, Mo, Ni, P, Pb, Sb, and Sr; all r ≥ 0.60, p < 0.05), but the correlation is insignificant for the other 6 elements (all r < 0.50, p > 0.05; Pearson correlation test; Fig. 2).

### Concentration difference

Differences in elemental concentrations between the two lichens (tested by Paired samples T test, α = 0.05) are presented in Table 1. These results show that PHh was similar to CAf in concentrations for 6 elements (Cd, Cr, K, Mo, P and Zn) but was higher than the latter for the other 14 elements (p < 0.05; Table 1). For brevity and clarity, these differences are also presented in Fig. 2.

Differences in elemental concentrations among the 4 plots (P1, 5–10 m off the road; P2, 100 m; P3, 200 m; and P4, 400 m) were tested by Paired samples T test followed by Bonferroni correction (α = 0.0083), and illustrated using heatmap analysis on the range-standardised concentrations (standardised according to Equation 2). These results show that G3 elements were highest near the road, whereas G4 elements (K, P, and Mg) showed an inverse pattern in all lichen data sets (Figs 2 and 3). The difference was insignificant for 5 metals (Na, V, Ti, Cu, and Zn) and significant for 2 metals (Co and Cr) in all three data sets (Figs 2 and 3). A significant difference was found for 7 elements (K, Mg, Mn, Mo, Ni, P, and Pb) in both LC and CAf data sets, 2 metals (Ca and Sr) in both LC and PHh data sets, 2 metals (Fe and Sb) in the LC data set, Cd in the PHh data set, and Ba in the CAf data set.

## Discussion

### Soil contribution and road effects

Generally, our results show that all studied elements, barring Cd and Zn, are mainly of natural terrigenous in origin and soil contribution to lichen elemental burden is partly influenced by the road.

The high importance of SS on lichen elemental burdens can be inferred by the low EF_SS_ (<1.8) of G3 and G4 metals (Table 1; Fig. 2). Because an EF of <5 is often evidence of crustal input^5, 32^, all these elements are mainly of crustal origin. This conclusion is supported by the Fe:Ti ratios, which if similar between the lichen and soil samples, indicate entrapment of coarse soil particles in lichen thalli^3, 28, 35^. Fe and Ti are highly positively correlated in all lichen and SS data sets (all r > 0.979, p < 0.001), and the Fe:Ti ratios are not significantly different between lichens and SS (Table 1). Because a good positive correlation between lichen elements can be evidence of a common origin^11, 32, 34^, the intimate correlations between G3 metals and between G4 elements in lichens is further evidence of terrigenous origin, although these correlations are also a reflection of similar spatial patterns (Figs 2 and 3). Actually, most G3 and G4 elements, such as Fe, K and Ti, are regarded as of crustal origin in Inner Mongolia^32^, even in the Taihang Mountains that experiences heavy air pollution^31, 33, 34^. Although P had an EF_SS_ of >10 in the lichens (Table 1), its significant positive correlation with K (r > 0.790, p < 0.005) and Mg (r > 0.590 and p < 0.005) in the three data sets of lichens (Figs 2 and 3) suggests that the P enrichment in lichens is most likely a result of active absorption and/or bio-regulation of this essential nutrient rather than evidence of anthropogenic input. Anthropogenic P emission is less probable because production and use of P-enriched fertilisers/pesticides has been negligible in Duolun County.

The high importance of soil contribution to lichen elemental composition is also inferred by the source of Cu. The poor correlation of Cu with terrigenous elements (G3 and G4 elements; Figs 2 and 3) may be partly explained by the fact that Cu is often associated with industrial activities and road traffic^3, 11^. A high EF_SS_ of Cu in lichens is expected because samples were collected within 400 m off the road. However, EF_SS_ of Cu is close to the unity with a low variation (CV <21%; Table 1), indicating a predominant terrigenous input superimposed on the road effects.

The high contribution of soil input is clearly due to deposition of wind-blown soil particles on lichen thalli. It is well known that most steppes have been heavily degraded/desertified during the last several decades in the region. The nearest sand dune complex is only 2.5 km north of the sampling sites (Fig. 1). Surface soil in these ecosystems is vulnerable to wind erosion and serves as an important source of frequent sand storms. Atmospheric deposition of these soil particles is a predominant source of lichen elements, as suggested in our recent study in Xilin River Basin, 200 km north of the study area^32^.

The soil contribution to lichen elemental burden appears to have been influenced by road traffic. Concentrations of G3 metals were highest in plots P1 in all three data sets, although multicomparison analyses show that the difference is insignificant for 3 metals (Na, V and Ti) and significant for only 2 metals (Co and Cr) in all three data sets (Figs 2 and 3). Nevertheless, a decreasing trend in concentration of most terrigenous metals with increasing distance from the road can be observed in the plots 5–200 m off the road. The presence of such a pattern has been shown by many relevant studies^3^ and is probably due to deposition of terrigenous particles raised by vehicle traffic on lichen thalli. G4 elements show an inverse pattern, characterised by the highest concentrations in plots P4, although the difference is not significant in the PHh data set. This pattern may be a result of better lichen physiology because the three elements are all essential nutrients for plants.

A comparison with relevant studies shows that soil input has greatly increased lichen elemental concentrations in the study area. Concentrations of all terrigenous elements, barring Ca and Mg, are generally higher than or similar to high literature value ranges from various lichens in diverse ecosystems. For example, concentrations of Fe, Ti, and V are often 2–75-fold, 1.6–55-fold and 2–45-fold literature values outside North China^1, 2, 6–29, 35–40^, respectively. The three metals are similar in concentration to those reported from North China^31–34^. Concentrations of 7 elements (Ba, Cu, K, Na, P, Pb, and Sb) are in the range of the values from Xilin River Basin^32^. Lichens from the Taihang Mountains, with heavy air pollution, are similar in concentration for most of these elements but are higher for Ca, Mg and Pb^34^.

### Anthropogenic input and road effects

The results show that both Cd and Zn were most likely of anthropogenic origin, and concentration of both metals changed little with distance from the road.

The anthropogenic origin of both Cd and Zn can be inferred from their EF_SS_ > 5.0 (Table 1), which is most likely an indication of a non-crustal or non-local source input^5, 32^. The poor correlations of both metals with terrigenous elements also support this inference (Figs 2 and 3). The same result has been observed in a similar ecosystem of the Xilin River Basin, where high EF_SS_ for Cd and Zn and their poor correlation with other elements were attributed to road emissions^32^.

It is known that Cd and Zn are traffic-related metals and often accumulate in lichens near roads^3^. However, a decreasing concentration trend with increasing distance from the road was not observed for Cd and Zn. Actually, concentrations of both metals were rather consistent among plots (Figs 2 and 3) and had a low variability (CV < 11% in all three data sets of lichens; Table 1). This pattern might be due to two sources of input superimposed on the wind-blown soil particle deposition. First, these metals may be emitted from vehicles with a dispersion distance exceeding 400 m, as shown by the enrichment of both metals in lichens 10 km off a highway in the Xilinhot River Basin^32^. Another possible source may be coal combustion in Dolon Nor town, 5 km north of the study sites. The town had a population of approximately 30,000 inhabitants at the time of collection. Coal has been used for many years for domestic heating during the seven colder months (from Oct. 1 to May 1), and released metals could be transported to the lichen thalli with prevailing southward wind in these months. The coal chemical industry (12 km NE of the study sites) and railway (established in 2008, for transportation of coal to industry) are unlikely significant contributors because the industrial operation was not started until September, 2011.

A comparison with other studies shows that Cd and Zn concentrations in our lichens are at the high literature value range or 2–6-fold documented concentrations^1, 11–28, 35, 37–40^. Only a handful of studies, mostly conducted in industrial or urban regions, have documented higher concentrations of Cd and Zn, which are often 1.5-8-fold our data^7–10^. These results suggest a high anthropogenic input in the region.

### Species difference

It is known that elemental composition in lichens are species- and element-specific^3, 32, 33^. This pattern is shown in our study. An insignificant difference between CAf and PHh for concentrations of 6 elements (Cd, Cr, K, Mo, P, and Zn; Table 1, Fig. 2) suggests that the two lichens are comparable in monitoring these elements. Concentrations of the other 14 metals were higher in PHh than in CAf (p < 0.05; Table 1, Fig. 2). A lack of good positive correlation between CAf and PHh in concentration of 4 metals (Na, Ba, V and Ti; r < 0.50, p > 0.05; Fig. 2) indicates that the two lichens are not substitutes for each other in monitoring atmospheric deposition of these metals. In contrast, concentration of 10 metals (Ca, Co, Cu, Fe, Mg, Mn, Ni, Pb, Sb, and Sr) in one lichen can be calculated from the other by linear regression analysis, as indicated by the high positive correlation between the two lichens (r ≥ 0.65, p < 0.01; Fig. 2).

Our results show that lichen elemental compositions in the study area are the result of both natural occurrence and anthropogenic input. Wind-blown dust represents a predominant source of lichen elements, and road traffic can enhance lichen elemental burden by increasing emission of soil particles. Anthropogenic emissions from the town and road traffic have also led to the enrichment of Cd and Zn in lichens. Although PHh was higher than CAf in concentrations of 14 terrigenous metals, both lichens are applicable in biomonitoring atmospheric element deposition and in most cases yield comparable results.

## Methods

### Study area

The study was conducted in Duolun County, Inner Mongolia, North China. Duolun County is an agro-pastoral ecotone, 200 km north of Beijing (Fig. 1). Dolon Nor town (42°12′N, 116°29′E) is the county seat of Duolun County, with a population of approximately 30,000 inhabitants in 2011. The climate is typical of the Inner Mongolian Plateau with low annual precipitation (380 mm, mostly occurring as rainfall during May-September) and drought and windy episodes during the winter-spring season. Prevailing winds in the area are from NW, W and N in winter and from SW and S in summer. The predominant ecosystems are steppes and sand dunes. Sand dust and fuel combustion are two main factors influencing air quality in the area. Dust storms have been frequent due to steppe degradation and desert expansion in the last several decades. A large amount of fuel has been used for traffic (primarily gasoline) and heating (primarily coal) as the heating season lasts about seven months (from Oct. 1 to May 1). Chemical fertilisers and pesticides have been rarely used, and range fires have been very rare. There were very low, if any, industrial activities prior to 2011. A coal chemical industry was established in 2011, and a railway for transportation of coal to support industry was established in 2008. However, the industrial operation of the coal chemical industry was not started until September 2011.

In August 18, 2011, samples were collected in a *Populus* forest. The forest is 5 km south of Dolon Nor town, 2.5 km south of a sand dune complex, and 12 km SW of coal chemical industry (Fig. 1). The forest was dominated by *P*. *cathayana* and *P*. *davidiana*, planted during 1980’s and 1990’s. The terrain is rather flat with an elevation of 1,268–1,274 m.

### Sampling strategy

Three sampling lines, perpendicular to a north-south provincial road crossing the forest, were set at an interval of 800 m. At each sampling line, 4 plots 10 × 10 m^2^ in size at a distance from the road of 5–10, 100, 200 and 400 m were selected. At each plot, two epiphytic lichens (PHh and CAf) were collected at a height of 1.0–2.0 m above ground from 5–10 trees to obtain 15–30 thalli and stored in paper bags. These species were selected because they are dominant in the sampling area. To collect soil samples, 3 plots 50 × 50 m^2^ in size were randomly selected along each sampling line. At each plot, 10 SS samples (0–10 cm) were randomly collected using a soil gauge, mixed to represent average element composition, and stored in polyethylene bags.

### Sample preparation and chemical analysis

Lichen thalli were carefully cleaned under a microscope, and then were oven-dried at 70 °C for 72 h to a constant weight. Samples were not washed before mineralisation to avoid the leaching of ionisable forms or loss of material, as suggested by the relevant studies^6, 32, 34^. SS samples were thoroughly cleaned and dried using the above method. All samples were ground and homogenised using a grinding mill equipped with Tungsten Carbide jars (Retsch MM400; Retsch GmbH, Haan, Germany).

Samples (200–300 mg) were mineralised in a mixture of HNO_3_ and H_2_O_2_ for lichens, and in a mixture of HNO_3_, HF, HCl, and HClO_4_ for SS samples. The concentration of 20 elements (Ba, Ca, Cd, Co, Cr, Cu, Fe, K, Mg, Mn, Mo, Na, Ni, P, Pb, Sb, Sr, Ti, V and Zn) was determined using an inductively coupled plasma mass spectrometry (ICP-MS; Agilent 7700X; Agilent Technologies, Tokyo, Japan) at the Hebei Geological Laboratory. Analytical quality control was assured using a series of standard reference materials: IAEA-336 (Portuguese Lichen, issued by the International Atomic Energy Agency), GBW10014 (cabbage), GBW10015 (spinach) and GBW10052 (green tea) for lichens, as well as GBW07451, GBW07452 and GBW07457 for SS (all materials mentioned above were issued by the Institute of Geophysical and Geochemical Exploration, Chinese Academy of Geological Sciences). Methods have been published elsewhere^31, 32, 34, 41^.

### Calculation of EF_SS_

EF_SS_ values were calculated according to Equation (1).

1 $${{\rm{EF}}}_{{\rm{SS}}}=\frac{{([{\rm{El}}]/[{\rm{Fe}}])}_{{\rm{Lichen}}}}{{([{\rm{El}}]/[{\rm{Fe}}])}_{{\rm{SS}}}}$$

where “Fe” and “El” are the chosen reference element (Fe) and the element under consideration, respectively. The subscripts and the square brackets denote the sample type and concentrations, respectively.

### Range-standardisation of elemental concentration

The raw concentration was range-standardised according to Equation (2) prior to producing the heatmap.

2 $$[{\rm{El}}]\text{'}=\frac{([{\rm{El}}]-{[{\rm{El}}]}_{{\rm{\min }}})}{({[{\rm{El}}]}_{{\rm{\max }}}-{[{\rm{El}}]}_{{\rm{\min }}})}$$

where “[El]” and “[El]′” denote the raw elemental concentration and the range-standardised values, respectively. The subscripts “min” and “max” denote minimum and maximum raw concentrations, respectively.

### Statistical analyses

A one-sample Kolmogorov-Smirnov test was performed on the raw concentration distribution of each element. Because of the normal data distribution, parametric analyses were used in subsequent analyses. Concentration correlations between lichen elements were tested using a Pearson correlation analysis and cluster analysis. The cluster analysis was performed using the UPGMA criterion to construct the hierarchical tree and correlation distance to measure dissimilarities. The heatmap was produced on the range-standardised concentrations. All statistical analyses mentioned above were performed using the Past 3.14 software (Ø. Hammer, Sep. 2016).

Concentration differences between lichen species and among plots were tested using Paired-samples T test on the raw concentrations. Bonferroni corrections were used in the case of multicomparison. These analyses were performed using SPSS 13.0 (SPSS Inc., Chicago, IL, USA). Diagrams in Fig. 1 were prepared using Inkscape software 0.91 (Free Software Foundation Inc., USA; http://www.inkscape.org/).

## References

[1] Achotegui-Castells A, Sardans J, Ribas À, Peñuelas J (2013). Identifying the origin of atmospheric inputs of trace elements in the Prades Mountains (Catalonia) with bryophytes, lichens, and soil monitoring. Environ. Monit. Assess..

[2] Giordano S, Adamo P, Sorbo S, Vingiani S (2005). Atmospheric trace metal pollution in the Naples urban area based on results from moss and lichen bags. Environ. Pollut..

[3] Garty J (2001). Biomonitoring atmospheric heavy metals with lichens: theory and application. Crit. Rev. Plant Sci.

[4] Smodiš B (2004). Validation and application of plants as biomonitors of trace element atmospheric pollution – a co-ordinated effort in 14 countries. J. Atmos. Chem..

[5] Nash TH, Gries C (1995). The use of lichens in atmospheric deposition studies with an emphasis on the Arctic. Sci. Total Environ..

[6] Bari A, Rosso A, Minciardi MR, Troiani F, Piervittori R (2001). Analysis of heavy metals in atmospheric particulates in relation to their bioaccumulation in explanted *Pseudevernia furfuracea* thalli. Environ. Monit. Assess..

[7] Bajpai R, Upreti D, Dwivedi S (2010). Passive monitoring of atmospheric heavy metals in a historical city of central India by *Lepraria lobificans* Nyl. Environ. Monit. Assess..

[8] Bennett JP, Wright DM (2004). Element content of *Xanthoparmelia scabrosa* growing on asphalt in urban and rural New Zealand. Bryologist.

[9] Demiray AD, Yolcubal I, Akyol NH, Çobanoğlu G (2012). Biomonitoring of airborne metals using the lichen *Xanthoria parietina* in Kocaeli Province, Turkey. Ecol. Indic..

[10] Kłos A, Rajfur M, Wacławek M (2011). Application of enrichment factor (EF) to the interpretation of results from the biomonitoring studies. Ecol. Chem. Eng. S.

[11] Agnan Y, Séjalon-Delmas N, Probst A (2013). Comparing early twentieth century and present-day atmospheric pollution in SW France: a story of lichens. Environ. Pollut..

[12] Balabanova B, Stafilov T, Sajn R, Baceva K (2012). Characterisation of heavy metals in lichen species *Hypogymnia physodes* and *Evernia prunastri* due to biomonitoring of air pollution in the vicinity of copper mine. Int. J. Environ. Res..

[13] Cayir A, Coskun M, Coskun M (2007). Determination of atmospheric heavy metal pollution in Canakkale and Balikesir Provinces using lichen (*Cladonia rangiformis*) as a bioindicator. Bull. Environ. Contam. Toxicol..

[14] Chiarenzelli J (2001). Multi-element and rare earth element composition of lichens, mosses, and vascular plants from the Central Barrenlands, Nunavut, Canada. Appl. Geochem..

[15] Conti ME, Pino A, Botrè F, Bocca B, Alimoniti A (2009). Lichen *Usnea barbata* as biomonitor of airborne elements deposition in the Province of Tierra del Fuego (southern Patagonia, Argentina). Ecotox. Environ. Safe..

[16] Darnajoux R, Lutzoni F, Miadlikowska J, Bellenger J-P (2015). Determination of elemental baseline using peltigeralean lichens from Northeastern Canada (Québec): Initial data collection for long term monitoring of the impact of global climate change on boreal and subarctic area in Canada. Sci. Total Environ..

[17] Gandois L (2014). Use of geochemical signatures, including rare earth elements, in mosses and lichens to assess spatial integration and the influence of forest environment. Atmos. Environ..

[18] Loppi S, Pirintsos SA (2003). Epiphytic lichens as sentinels for heavy metal pollution at forest ecosystems (central Italy). Environ. Pollut..

[19] Mitrović T (2012). Epiphytic lichen *Flavoparmelia caperata* as a sentinel for trace metal pollution. J. Serb. Chem. Soc..

[20] Nimis P, Lazzarin G, Lazzarin A, Skert N (2000). Biomonitoring of trace elements with lichens in Veneto (NE Italy). Sci. Total Environ.

[21] Pacheco AM, Freitas M (2004). Are lower epiphytes really that better than higher plants for indicating airborne contaminants? An insight into the elemental contents of lichen thalli and tree bark by INAA. J. Radioana. Nucl. Ch.

[22] Riga-Karandinos AN, Karandinos MG (1998). Assessment of air pollution from a lignite power plant in the plain of Megalopolis (Greece) using as biomonitors three species of lichens; impacts on some biochemical parameters of lichens. Sci. Total Environ..

[23] Rizzio E, Bergamaschi L, Valcuvia MG, Profumo A, Gallorini M (2001). Trace elements determination in lichens and in the airborne particulate matter for the evaluation of the atmospheric pollution in a region of northern Italy. Environ. Int..

[24] Scerbo R (2002). Lichen (*Xanthoria parietina*) biomonitoring of trace element contamination and air quality assessment in Pisa Province (Tuscany, Italy). Sci. Total Environ..

[25] Singh SM (2013). Atmospheric deposition studies of heavy metals in Arctic by comparative analysis of lichens and cryoconite. Environ. Monit. Assess..

[26] Valeeva EI, Moskovchenko DV (2002). Trace-element composition of lichens as an indicator of atmospheric pollution in northern West Siberia. Polar Geogr.

[27] Varrica D, Aiuppa A, Dongarrà G (2000). Volcanic and anthropogenic contribution to heavy metal content in lichens from Mt. Etna and Vulcano island (Sicily). Environ. Pollut..

[28] Vieira BJ (2004). Element-enrichment factors in lichens from Terceira, Santa Maria and Madeira Islands (Azores and Madeira Archipelagoes). J. Atmos. Chem..

[29] Jeran Z, Jaćimović R, Batič F, Mavsar R (2002). Lichens as integrating air pollution monitors. Environ. Pollut..

[30] Sujetoviene G (2010). Road traffic pollution effects on epiphytic lichens. Ekologija.

[31] Zhao LC (2016). Optimization of ICP-AES and ICP-MS techniques for the determination of major, minor and micro elements in lichens. Spectrosc. Spect. Anal.

[32] Liu HJ (2016). Lichen elemental composition distinguishes anthropogenic emissions from dust storm input and differs among species: evidence from Xilinhot, Inner Mongolia, China. Sci. Rep.

[33] Liu HJ (2016). Effects of species and substrate preference on element concentration in six lichens in Taihang Mountains, Hebei, China. Mycosystema.

[34] Liu HJ (2016). Use of the lichen *Xanthoria mandschurica* in monitoring atmospheric elemental deposition in the Taihang Mountains, Hebei, China. Sci. Rep.

[35] Bennett JP, Wetmore CM (2000). 16-Year trends in elements of lichens at Theodore Roosevelt National Park, North Dakota. Sci. Total Environ..

[36] Dongarrà G, Varrica D (1998). The presence of heavy metals in air particulate at Vulcano island (Italy). Sci. Total Environ..

[37] Loppi S, Pirintsos SA, De Dominicis V (1999). Soil contribution to the elemental composition of epiphytic lichens (Tuscany, Central Italy). Environ. Monit. Assess.

[38] Scerbo R (1999). Lichen (*Xanthoria parietina*) biomonitoring of trace element contamination and air quality assessment in Livorno Province (Tuscany, Italy). Sci. Total Environ..

[39] Zhang ZH, Chai ZF, Mao XY, Chen JB (2002). Biomonitoring trace element atmospheric deposition using lichens in China. Environ. Pollut..

[40] Bajpai R, Mishra S, Dwivedi S, Upreti DK (2016). Change in atmospheric deposition during last half century and its impact on lichen community structure in Eastern Himalaya. Sci. Rep.

[41] Zhao LC (2017). Determination of elemental concentrations in lichens using ICP-AES/MS. Bio-protocol.

